# Traumatic ulna fracture with plastic deformation of the radius in a skeletally mature pregnant female: a case report

**DOI:** 10.1007/s00590-025-04350-0

**Published:** 2025-06-04

**Authors:** Zachariah Whiting, Austin Williams, Robert Wetzel

**Affiliations:** University Hospitals of Cleveland, Cleveland, USA

**Keywords:** Plastic deformation, Osteotomy, DRUJ, Ulna fracture, Pregnancy

## Abstract

**Case:**

A 20-year-old pregnant female presented with an ulna fracture after a crush and bending type injury. Anatomic reduction of her ulna was not possible due to plastic deformation of the radius, requiring a corrective osteotomy of the radius. She is now 16 months out from surgery with full painless range of motion and radiographic union.

**Conclusion:**

Plastic deformation of forearm fractures in adults is a rare phenomenon. It is possible that the patient’s pregnancy contributed to her injury. Patients with uncommon fracture morphology can make operative intervention more complicated and necessitate additional procedures to achieve optimal motion and alignment.

## Introduction

Plastic deformation of forearm fractures is a common occurrence in the pediatric population resulting from the complex molecular nature of pediatric bone. Pediatric cortical bone has a lower mineral content than adult bone, resulting in different material properties. Pediatric bone has a lower modulus of elasticity and less mineralization [1]. The increased plasticity allows pediatric long bones to absorb more energy, resulting in bending prior to fracture [2]. In contrast, plastic deformation occurs infrequently in adults. With increasing age, there are a decreasing proportion of circumferential lamellar bone, increasing number of osteons, increasing mineralization, and decreasing flexibility [3].

Whereas most adult forearm fractures result from higher-energy trauma, plastic deformations typically result from low-energy trauma with slow, constant bending forces. This is oftentimes seen in the setting of injuries involving industrial machinery, such as conveyor belts [4–6]. Patients will present with pain, potentially visible forearm deformities, and limitations with pronation and supination. Unfortunately, these injuries are frequently missed. Additionally, it is important to assess for injuries to the distal and proximal radioulnar joints, as these injuries can affect treatment and outcomes.

Pediatric patients with plastic deformities have significant potential for correction with growth. Adults on the other hand can be severely disabled due to restrictions with pronation and supination. Failure to restore the anatomic radial bow to within 4 to 5% of the contralateral side has been associated with up to a 20% loss of forearm rotation [7]. Closed management can be attempted, but surgical intervention in the form of osteotomies with open reduction internal fixation (ORIF) is indicated if closed reduction is unsuccessful or if the patient has any range of motion (ROM) deficits.

## Case report

**Fig. 1:**
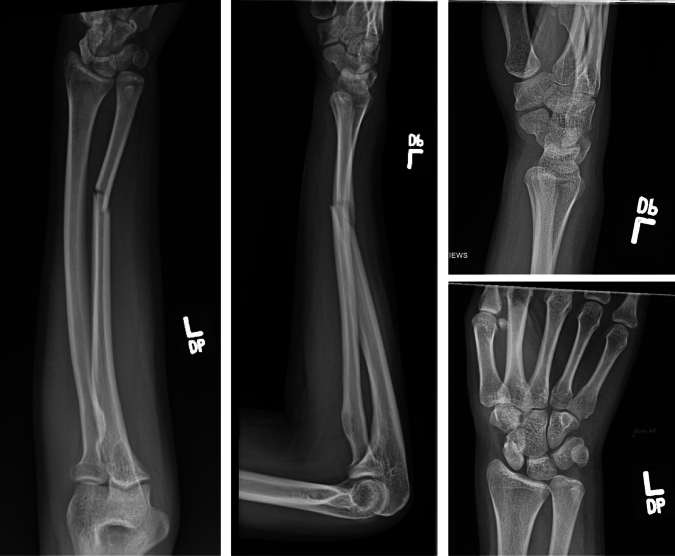
Injury radiographs demonstrating a displaced and translated midshaft ulna fracture, concern for DRUJ injury, and positive ulnar variance

**Fig. 2:**
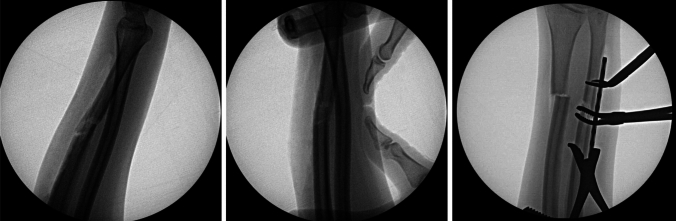
Intraoperative radiographs demonstrating plastic deformation of the radius, unsuccessful closed osteoclasis, and radial shaft osteotomy

This is the case of a 20-year-old left-hand-dominant female who suffered a left ulna fracture with associated distal radioulnar joint (DRUJ) injury after her arm was caught in an industrial laundry folding machine at work. Her past medical history was only notable for being 14 weeks pregnant. On examination, she was closed and neurovascularly intact. She had very limited pronation and supination and her forearm was held in a semi-supinated position. Radiographs revealed a displaced and translated midshaft ulna fracture and concern for DRUJ injury (Fig. 1). As a result of her injury, she was indicated for operative fixation. Intraoperatively, the ulna was able to be anatomically reduced with pointed reduction clamps. However, the DRUJ remained dislocated with an extremely positive ulnar variance. The DRUJ remained dislocated in neutral, pronation, and supination. We checked the contralateral DRUJ to evaluate her baseline stability and her injured DRUJ had more motion than the contralateral uninjured DRUJ. At this point, it was obvious that the plastic deformation had changed the bow of the radius. Closed osteoclasis was unsuccessfully attempted (Fig. 2). Her deformity was defined as an apex volar and radial bend with a radial bow of 14.9 mm. It was then decided that a radial shaft osteotomy would be necessary to correct both planes of deformity, provide length, and restore her function.

**Fig. 3:**
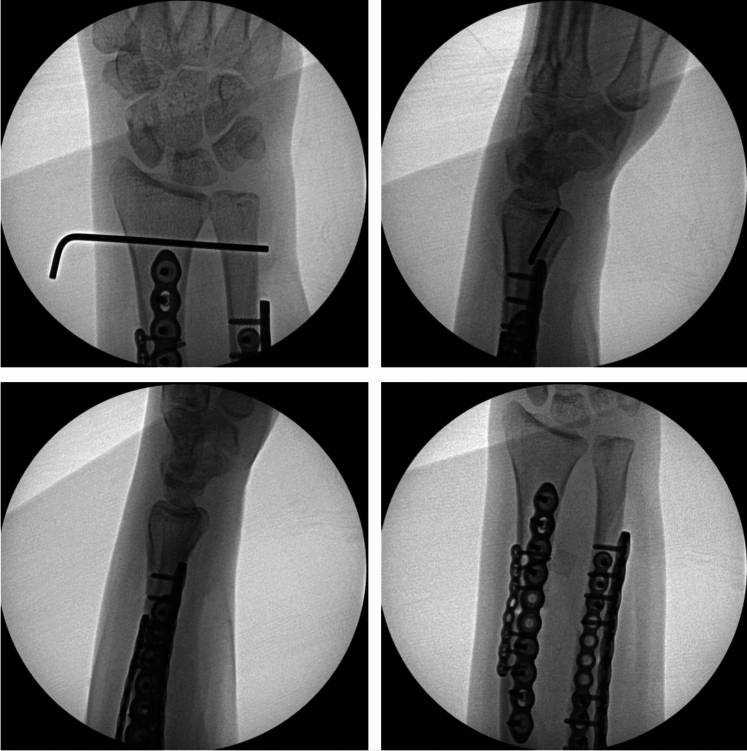
Intraoperative radiographs demonstrating reduced DRUJ and neutral ulnar variance after radial osteotomy and fixation of both the radius and ulna

**Fig. 4:**
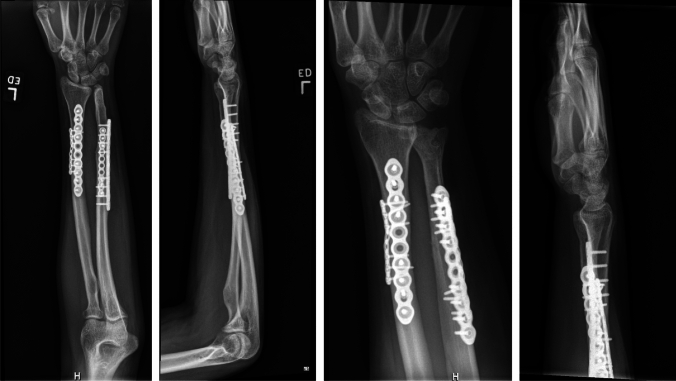
Postoperative radiographs at 16 months demonstrating maintenance of reduction and osseous union

**Fig. 5:**
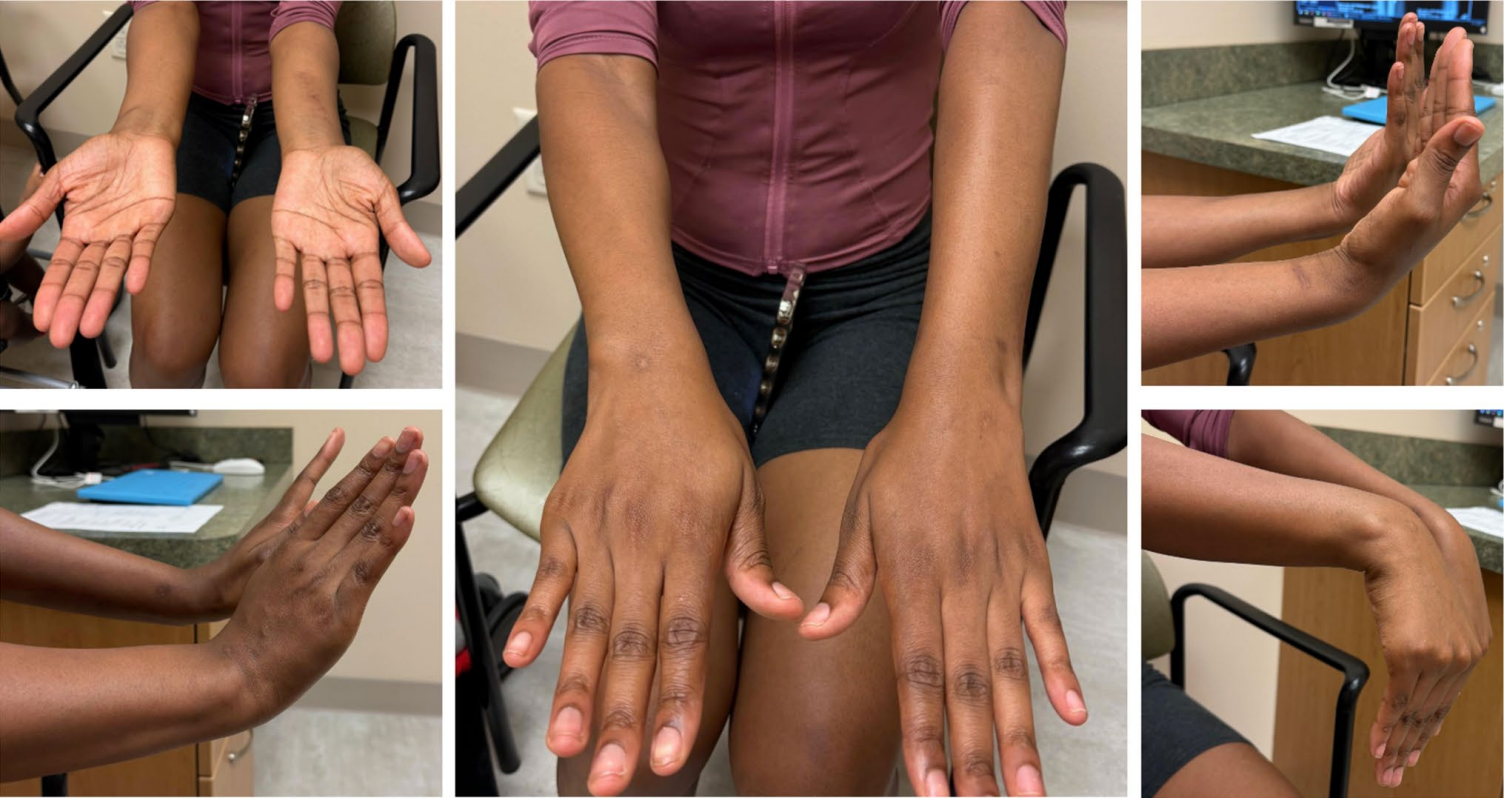
Postoperative clinical photos at 16 months demonstrating full active ROM symmetrical to her contralateral side

We chose to perform the radial shaft osteotomy at the level of the ulna fracture. This was where the bending force on the radius had occurred and thus was an appropriate site for osteotomy correction. By choosing this site for our osteotomy, we were able to create a large degree of correction. Multiple drill holes were made, and an osteotomy was performed (Fig. 2). A mini fragment plate was then contoured to correct the plastic deformities. Radial bow was corrected to 11.6 mm, and radial length was restored. The ulna was able to be anatomically reduced and an ORIF was performed. With the apex volar ulnar deformity corrected, the DRUJ became quite stable with neutral variance. Given the instability due to the injury, the DRUJ was stabilized with percutaneous K-wire fixation (Fig. 3). She was placed in a sugar-tong splint in neutral rotation and remained non-weight-bearing for 6 weeks until her DRUJ fixation was removed in the office setting. Interval radiographs at each postoperative visit showed maintenance of reduction of the radius, ulna, and DRUJ with bony healing. She is now over 16 months out from surgery with radiographic union of both her ulna fracture and radius osteotomy (Fig. 4). She has full painless ROM which is symmetrical to her non-injured contralateral side (Fig. 5). Consent to publish data concerning this case was obtained from the patient presented in the current study.

## Discussion

Plastic deformation of forearm fractures is an incredibly rare and oftentimes missed injury in the adult population. Despite its rarity, it can have detrimental consequences to patient outcomes and function. Our patient had an obvious ulna fracture with subtle radial plastic deformation. There was also a subtle DRUJ injury on preoperative radiographs. Intraoperatively, the case quickly became complex as a corrective osteotomy of the radius was required to anatomically reduce both the ulna and DRUJ.

Over the years, there have been several case reports about plastic deformation of forearm fractures in adults. Tada et al. presented two cases of young adults with plastic deformations of forearm fractures. One patient underwent ORIF of a radius fracture, and ulnar bowing was noted on postoperative radiographs. The patient was pain-free with adequate ROM, and thus, no additional intervention was undertaken. The other patient underwent osteotomy and ORIF of her ulna, but closed management of her radius because full ROM was noted intraoperatively [8].

Moon et al. presented two cases of young women with plastic deformations of forearm fractures as a result of injuries sustained on conveyor belts. Both patients required osteotomies to anatomically reduce their fractures [5]. When Moon et al. published in 2011, they found 17 cases of adult forearm plastic deformation reported in the literature. The average patient age was 21.7 years, with a range from 18 to 30 years. In 15 of 17 cases, the mechanism was a gradual compression or crush from a transverse force exerted by industrial equipment. Four of 17 patients underwent surgical management of their injuries with good clinical and radiographic results [5].

Tianhao et al. retrospectively reviewed 30 cases of plastic forearm deformations in adults. Average age was 21.3 years. 96.7% of patients suffered injuries when their arm was entrapped in a machine with moving rollers. Thirteen patients underwent surgical intervention with all patients achieving adequate forearm ROM [4].

Our patient was a young female who also suffered a gradual compression force from a laundry folding machine. Her mechanism of injury is consistent with documented instances of plastic deformation in young adults. Incidentally, she was also noted to be pregnant at the time of her injury. Pregnancy results in a temporary decrease in bone density and mineralization [9, 10]. This decreased mineralization would, in theory, make bones during pregnancy more flexible and more similar to pediatric bones. There are several cases of plastic deformation occurring in young adults who are not pregnant, but the fact that our patient was pregnant at the time of injury and surgery may have made her bones more flexible. Alterations to her bone chemistry as a result of her pregnancy could have contributed to her having a plastic deformation of her radius, rather than a complete fracture.

In terms of our patient’s management, a closed osteoclasis of her radius was attempted after reduction of her ulna with pointed reduction clamps. Unfortunately, we were not able to reduce her radius with closed osteoclasis alone. If we had been successful and the DRUJ had reduced as well, we would have performed an isolated ORIF of her ulna. However, a corrective osteotomy of the radius was necessary to anatomically reduce both the ulna and DRUJ.

In adults, optimal treatment for forearm plastic deformation is unknown due to the rarity of these injuries. Closed reduction can be attempted and can result in adequate outcomes if the deformity is corrected and the patient achieves full ROM. However, if closed reduction is unsuccessful or if there are persistent deficits in pronation or supination, surgical correction with osteotomies is indicated.

## Data Availability

No datasets were generated or analyzed during the current study.

## References

[1] Currey J, Butler G (1975) The mechanical properties of bone tissue in children. J Bone Jt Surg 57(6):810–814 1158919

[2] Mabrey J, Fitch R (1989) Plastic deformation in pediatric fractures: mechanism and treatment. J Pediatr Orthop 9(3):310–314 2723051

[3] Anderson IF, Hume KF, Chong AL (1994) Plastic bowing of the forearm in the more matore skeleton. Aust N Z J Surg 64(2):132–134 8291979 10.1111/j.1445-2197.1994.tb02162.x

[4] Tianhao W, Yueju L, Yingze Z, Xirui W (2014) Plastic deformation of the forearm in adults: an analysis of 30 cases. J Orthop Surg Res 9:117 25444518 10.1186/s13018-014-0117-0 PMC4266215

[5] Moon ES, Howlett J, Wiater BP, Trumble TE (2011) Treatment of plastic deformation of the forearm in young adults with double-level osteotomies: case reports. J Hand Surg Am 36(4):639–646 21353396 10.1016/j.jhsa.2010.11.042

[6] Lefaivre KA, Slobogean GP, O’Brien PJ (2007) Case report: plastic deformation of the forearm in an adult—treatment with multiple osteotomies. Clin Orthop Relat Res 462:234–237 17415003 10.1097/BLO.0b013e31805c7405

[7] Schemitsch EH, Richards RR (1992) The effect of malunion on functional outcome after plate fixation of fractures of both bones of the forearm in adults. J Bone Joint Surg Am 74(7):1068–1078 1522093

[8] Tada K, Ikeda K, Tsubouchi H, Tomita K (2008) Acute plastic bowing of the forearm in adults: a report of two cases. J Orthop Surg (Hong Kong) 16(2):241–242 18725680 10.1177/230949900801600222

[9] Watts NB, Binkley N, Owens CD et al (2021) Bone mineral density changes associated with pregnancy, lactation, and medical treatments in premenopausal women and effects later in life. J Women’s Heal 30(10):1416–1430 10.1089/jwh.2020.8989 PMC13174973 34435897

[10] Ünal M, Saçıntı KG, Sezgin EA (2025) Pregnancy- and lactation-related bone fragility: the hidden risk. Jt Dis Relat Surg 36(1):210–213 39719919 10.52312/jdrs.2024.1957 PMC11734854

